# Integrated Proteogenomic Characterization Identifies Breast Cancer Immune Subgroups and Subtype-Specific Therapeutic Strategies

**DOI:** 10.34133/research.1271

**Published:** 2026-05-12

**Authors:** Guozheng Li, Yihai Chen, Yong Liu, Lei Liu, Shuaitong Chen, Wantong Sun, Tianze Pang, Chao Fang, Bo Wei, Xin Zhang, Zihan Yang, Hao Li, Shiyao Sui, Xiaomei Li, Maopeng Yang, Changjun He, Bin Liu, Weiyang Tao, Shouping Xu

**Affiliations:** ^1^ Department of Breast Surgery, Harbin Medical University Cancer Hospital, Harbin 150040, China.; ^2^ Department of Thyroid Surgery, The Second Affiliated Hospital, Zhejiang University School of Medicine, Hangzhou, Zhejiang 310009, China.; ^3^ Department of Scientific Research, Mudanjiang Medical University, Mudanjiang, Heilongjiang 157011, China.; ^4^ LC-Bio Technology Co. Ltd., Hangzhou, Zhejiang 310000, China.; ^5^ Department of Medical Oncology, Harbin Medical University Cancer Hospital, Harbin, China.; ^6^ Department of Pathology, Shenzhen People’s Hospital, The Second Clinical Medical College of Jinan University, The First Affiliated Hospital of Southern University of Science and Technology, Shenzhen, China.; ^7^ Department of Thoracic Surgery, Harbin Medical University Cancer Hospital, Harbin, China; ^8^ Heilongjiang University of Traditional Chinese Medicine, Harbin, Heilongjiang 150040, China.; ^9^ Department of Breast Surgery, The First Affiliated Hospital of Harbin Medical University, Harbin, China.

## Abstract

Breast cancer remains the most common malignancy in women worldwide, and its marked heterogeneity continues to impede effective treatment. Immunotherapy has reshaped the therapeutic landscape, yet only a subset of patients achieves durable benefit, in large part because of immune and metabolic diversity within the tumor microenvironment. Posttranslational modifications are key regulators that couple cellular signaling to metabolic and immune homeostasis. Here, we generated a multiomics immune-metabolic atlas of breast cancer by integrating genomics, transcriptomics, proteomics, lactylomics, and phosphoproteomics from 115 tumor and 99 matched adjacent tissue samples. Using immune infiltration profiling, we defined 3 immune-related breast cancer subtypes with distinct metabolic states, posttranslational modifications cross-talk, and mutational landscapes. Integrated analyses revealed that the interplay between protein lactylation and phosphorylation orchestrates glycolytic reprogramming and immune modulation. Furthermore, we identified subtype-specific histone lactylation patterns and signaling rewiring associated with therapeutic response. This atlas provides a comprehensive framework for dissecting immune-metabolic regulation in breast cancer and yields molecular insights to guide subtype-specific precision immunotherapeutic strategies.

## Introduction

Breast cancer (BC) remains a leading cause of cancer-related mortality worldwide, with its clinical heterogeneity driven by complex interactions among genetic alterations, remodeling of the tumor microenvironment (TME), and metabolic adaptation [1]. This underscores the urgent need for more effective treatment strategies. Although immune checkpoint inhibitors (ICIs) targeting programmed cell death protein 1 (PD-1)/programmed cell death ligand 1 (PD-L1) have revolutionized the treatment of triple-negative BC (TNBC), objective response rates remain suboptimal (12% to 19%) [2,3]. Major resistance mechanisms include immunosuppressive TME features such as dysfunctional CD8^+^ T cell infiltration, M2 macrophage polarization, and metabolic competition driven by lactate acidosis and lipid accumulation [4–6]. Recent phase III trials (KEYNOTE-355 and IMpassion130) further underscore the need to define the molecular characteristics of BCs that are most likely to benefit from immunotherapy [7,8], thereby enabling more personalized clinical management.

Multiomics approaches have begun to elucidate the immune-metabolic cross-talk in BC. For example, proteogenomic analyses by Keren et al. [9] identified spatial coordination between glycolytic enzymes and PD-L1 expression in immune-excluded tumors, while single-cell metabolomics revealed that *TP53* mutations promote lactate-dehydrogenase-A-dependent suppression of CD8^+^ T cells [10]. Phosphoproteomic studies demonstrate that phosphatidylinositol 3-kinase (PI3K)–AKT activation rewires TME through hypoxia-inducible factor-1α (HIF-1α)-mediated vascular endothelial growth factor (VEGF) secretion [11], and histone lactylation has emerged as a novel epigenetic regulator linking Warburg metabolism to macrophage polarization [12]. Despite major advances in omics-based cancer biology, current studies often focus on single molecular layers, leaving the coordinated regulation among genetic alterations, posttranslational modifications (PTMs), and immune remodeling insufficiently understood.

To systematically dissect this complexity, we established a multidimensional atlas of BC by integrating genomic, transcriptomic, proteomic, lactylomic, and phosphoproteomic data. This resource delineates 3 proteome-defined immune subtypes with distinct metabolic dependencies and therapeutic vulnerabilities. By coupling PTM landscapes with immune infiltration patterns, our study provides a comprehensive view of immune-metabolic reprogramming in BC. Collectively, this work serves as a reference map for exploring PTM-governed immune heterogeneity and offers potential molecular entry points for optimizing immunotherapeutic strategies.

## Results

### The comprehensive landscape of BC based on the multiomics

**Fig. 1.**
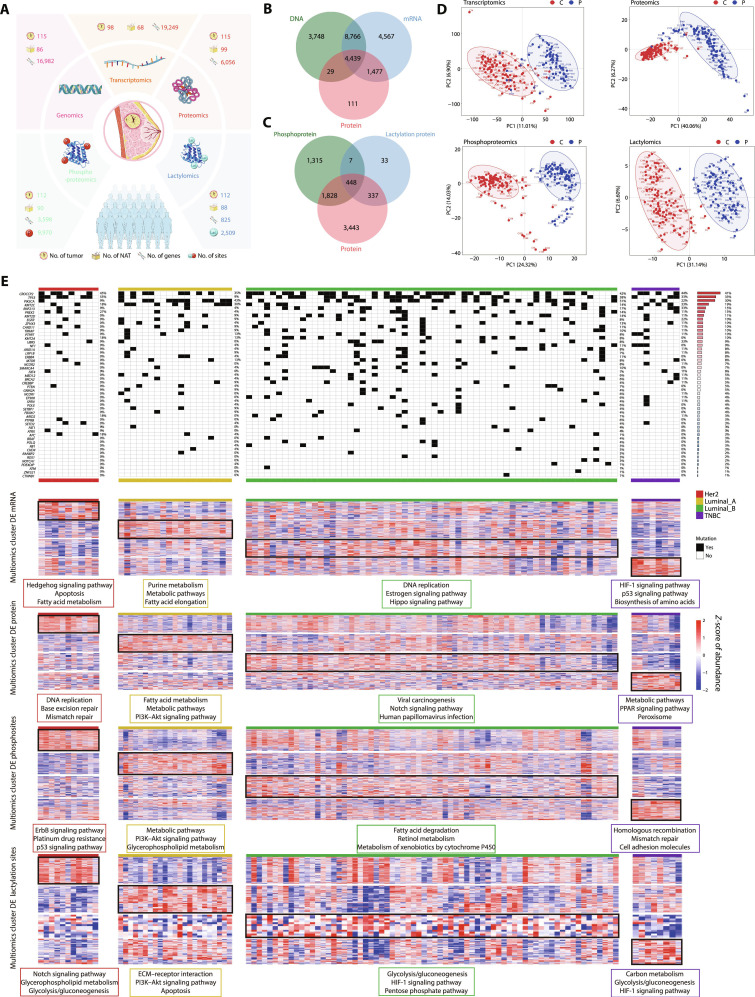
The comprehensive landscape of breast cancer based on the multiomics. (A) The overview of multiomics including (the number of samples, detected genes, and modification sites). (B) Venn plot showing the overlap of detected gene number in DNA, mRNA, and protein. (C) Venn plot showing the overlap of detected gene number in protein, phosphoprotein, and lactylation protein. (D) Partial least-squares discrimination analysis of the transcriptomics, proteomics, phosphoproteomics, and lactylomics. PC, principal component. (E) The heatmap shows the gene mutation frequency and differential gene and sites of 4 breast cancer subtypes. PPAR, peroxisome-proliferator-activated receptor.

We analyzed 115 treatment-naïve BC samples from Chinese patients, classified into human epidermal growth factor receptor 2 (HER2; *n* = 11), luminal A (*n* = 23), luminal B (*n* = 72), and TNBC (*n* = 9) subtypes (Table S1). The overall experimental design is summarized in Fig. 1A. Whole-exome sequencing (WES) was performed on all 115 tumors and 86 matched normal adjacent tissues (NATs), RNA sequencing (RNA-seq) on 98 tumors and 68 NATs, and proteomic profiling on all tumors and 99 NATs. PTMs were further characterized by phosphoproteomics (112 tumors and 90 NATs) and lactylomics (112 tumors and 88 NATs). In total, 16,982 DNA-level, 19,249 transcript-level, and 6,056 protein-coding genes were detected, of which only 4,439 were shared across all 3 omics layers (Fig. 1B). Phosphoproteomics and lactylomics identified 3,598 and 825 genes harboring phosphorylation and lactylation sites, respectively. Integrating these datasets with the proteome, we identified 448 genes jointly quantified at the protein, phosphorylation, and lactylation levels (Fig. 1C). Partial least-squares discriminant analysis further revealed greater intersample heterogeneity across all 4 omics in tumors than in NATs (Fig. 1D).

To delineate the basic molecular characteristics of these BC samples across the genomic, transcriptomic, proteomic, phosphoproteomic, and lactylomic layers, we profiled subtype-specific somatic mutations and compared gene expression and PTM levels among the 4 clinical subtypes (HER2, luminal A, luminal B, and TNBC). First, we calculated the mutation frequency of the 50 most frequently mutated genes in human cancers [13]. Luminal B BCs harbored the highest overall mutational burden among the 4 subtypes. *CROCCP2*, *TP53*, *PIK3CA*, *KMT2C*, *RNF213*, and *PREX2* exhibited high mutation frequencies across all subtypes. *ARID2*, *FAT1*, *ATRX*, and *APC* showed the highest mutation frequencies in HER2-positive BCs. Distinct groups of mutated genes were linked to different luminal BC subtypes: Mutations in *mTOR*, *SETBP1*, and *FBXW7* were predominantly observed in luminal A tumors, while *ERBB4*, *SMARCA4*, and *PTPRB* mutations were more commonly found in luminal B tumors. In TNBC, *BRCA2*, *NCOR1*, and *EP300* were the most frequently mutated genes (Fig. 1E). Functionally, HER2-associated differentially expressed (DE) proteins were enriched in DNA damage repair pathways, and HER2-associated DE phosphosites were enriched in *ErbB* and *p53* signaling pathways. In luminal A tumors, DE mRNAs, DE proteins, and DE phosphosites were predominantly enriched in metabolism-related signaling pathways. Luminal-B-associated DE lactylation sites were implicated in the HIF-1 signaling pathway and glycometabolism, whereas TNBC-specific DE lactylation sites showed similar enrichment in these processes (Fig. 1E).

### Proteomic intertumor immune heterogeneity and associated metabolic function in BC

**Fig. 2.**
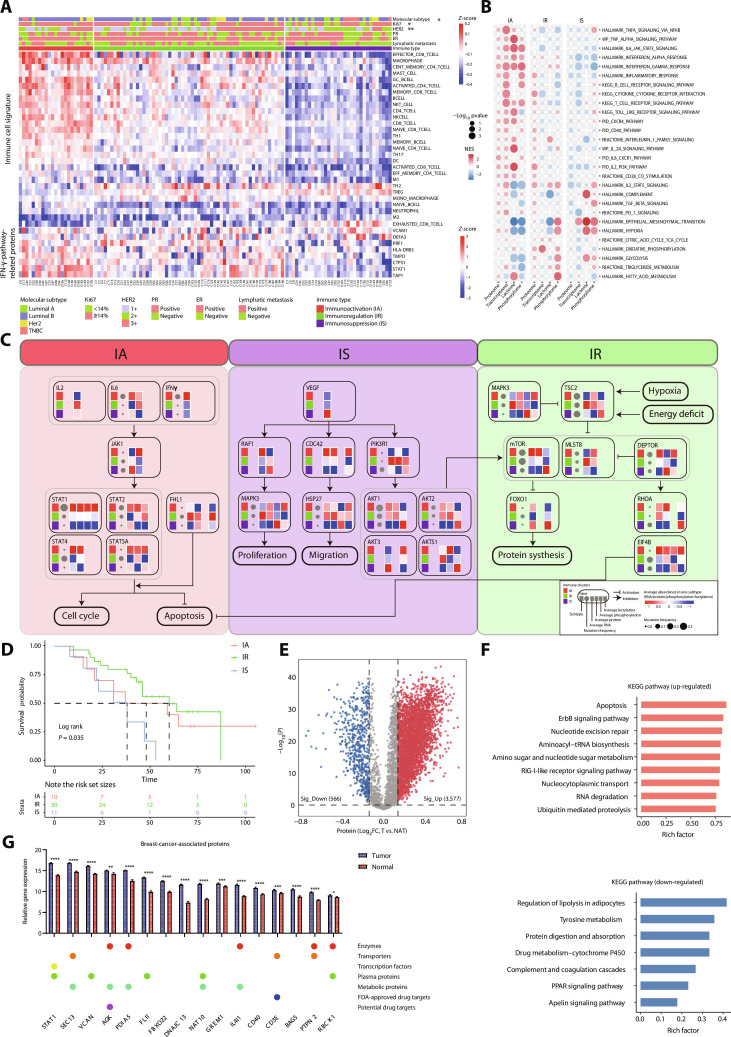
Proteomic intertumor immune heterogeneity and associated metabolic function in breast cancer. (A) According to the C7 (immunological signature gene sets) gene set of MSigDB (Molecular Signature Database), gene set variation analysis (GSVA) was performed, and relevant immune cell categories were screened for hierarchical clustering analysis (heatmap), showing 3 immune clusters. One-way ANOVA was performed to compare the 28 immune cell-type-related gene sets among different subtypes, followed by analysis of covariance (ANCOVA) with tumor purity included as a covariate to calculate adjusted *P* values and the expression levels of immune clusters related to IFN-γ pathway proteins. (B) Bubble plot representing MSigDB hallmark and canonical pathways enrichment among the 3 immune subtypes. (C) Three oncogenic pathways frequently altered in breast cancer. Each gene is annotated with mutational frequency, RNA, protein, phosphorylation, and lactylation modification abundance by 3 immune cluster. (D) Kaplan–Meier curves for disease-free survival of 3 immune clusters (log rank test). (E) Volcano plot depicting differentially expressed proteins between tumors and paired normal adjacent tissues (NATs) (*t* test). FC, fold change. (F) Kyoto Encyclopedia of Genes and Genomes (KEGG) pathways enrichment of up-regulated and down-regulated genes. (G) Box plot showing genes expression for breast-cancer-associated proteins annotated with potential clinical utilities by the Human Protein Atlas. FDA, Food and Drug Administration. *P < 0.05; **P < 0.01; ***P < 0.001; ****P < 0.0001.

Conventional molecular subtyping has long been used to guide BC treatment, but it provides limited guidance for immunotherapy. In this study, we aimed to delineate intertumor heterogeneity and characterize cellular metabolism across tumor immune subtypes in BC, considering that metabolic reprogramming is crucial for guiding immunotherapy strategies [14–16]. Immune-related signaling pathways derived from proteomics-based gene set variation analysis (GSVA) were used to estimate immune cell infiltration levels (Table S2). This analysis identified 3 proteome-defined immune subtypes: immunoactivation (IA), immunoregulation (IR), and immunosuppression (IS) (Fig. 2A). The IA subtype showed increased activity of immune-related pathways (Fig. 2B). We applied multiple approaches to validating this clustering: (a) Two cohorts from the Clinical Proteomic Tumor Analysis Consortium (CPTAC) database (PDC000173 and PDC000120) were classified using the same strategy and subjected to GSVA (Tables S3 and S4). The results were consistent with our subtype classification (Fig. S1A and C). Interferon-γ (IFN−γ) and other immune activation-related pathways were enriched in the IA subtype in the CPTAC cohorts. Moreover, the IS subtype in the CPTAC cohorts also exhibited high enrichment of hypoxia and glycolysis pathways (Fig. S1B and D). (b) xCell- and MCP-counter-derived cell type enrichment scores were used to verify immune cell infiltration across the 3 subtypes. CD4^+^ T helper 1 (Th1) cells, CD8^+^ T cells, natural killer (NK) cells, M1 macrophages, the overall immune score, and the cytotoxicity score were significantly elevated in IA tumors. IS tumors showed higher stromal scores and increased enrichment of endothelial cells, cancer-associated fibroblasts, and common myeloid progenitors (Fig. S1E). (c) Spearman correlation analysis showed that glycolysis and hypoxia pathways were negatively correlated with CD8^+^ T cells. In contrast, exhausted CD8^+^ T cells were positively correlated with these pathways (Fig. S1F and Table S5). (d) A Sankey diagram was used to illustrate the correspondence between the initial immune activity states (IA, IR, and IS) and downstream TME subtypes, including Depleted, Fibrotic, Immune_Enriched_Fibrotic, and Immune_Enriched_non_Fibrotic (Fig. S1G). Overall, distinct transition patterns were observed among the 3 initial states. Samples originating from the IA state predominantly transitioned toward the immune-enriched categories, indicating a tendency toward active microenvironments. The majority of IS samples flowed into Fibrotic and Depleted subtypes were observed. In addition, IR represents a transitional state with mixed characteristics. Univariate Cox regression analysis identified age (hazard ratio [HR] = 1.06, 95% confidence interval [CI]: 1.013 to 1.111, *P* = 0.0114) and HER2 positivity (HR = 2.25, 95% CI: 1.115 to 4.550, *P* = 0.0236) as significant risk factors for survival. Other clinicopathological variables, including lymph node status, histological grade, endoplasmic reticulum (ER) status, Ki67, and T/N stage, were not significantly associated with prognosis. Notably, immune typing was significantly correlated with survival. Compared with the IA subtype, the IS subtype exhibited a markedly increased risk of adverse outcome (HR = 5.12, 95% CI: 1.175 to 22.317, *P* = 0.0297), whereas no significant difference was observed for the IR subtype (*P* = 0.6078) (Fig. S1H).

To identify potential therapeutic targets for each immune subtype, we first assessed alterations in cancer-related signaling pathways among the 3 immune subtypes using gene set enrichment analysis (Fig. S1I to K). We then integrated genetic alterations with RNA, protein, phosphorylation, and lactylation data to interrogate 3 key oncogenic signaling pathways in BC: *JAK/STAT*, *mTOR*, and *VEGF/VEGFR* signaling (Fig. 2C). The *JAK* signaling pathway is significantly activated in the IA subtype, as reflected by high expression of *IL-6*, IFN−γ, and *STAT* family genes, along with increased RNA, protein, phosphorylation, and lactylation levels of *STAT1*, *STAT2*, *STAT4*, and *STAT5A*, indicating functional convergence at multiple molecular layers. In contrast, the IS subtype shows significant down-regulation of both expression and PTMs of multiple genes in this pathway. In the *mTOR* signaling pathway, IR tumors exhibit increased expression of *MLST8*, a core *mTOR* component, and decreased expression of *DEPTOR*, an *mTOR* inhibitory factor. Key protein-synthesis-related genes, *RHOA* and *EIF4B*, display lower lactylation levels, suggesting that high lactylation may inhibit their functions. Meanwhile, both expression and phosphorylation of the apoptotic gene *FOXO1* are markedly suppressed in the IR subtype, indicating inhibition of apoptosis in IR tumors. Within the VEGF pathway, IS tumors show higher expression of *RAF1*, which may promote tumor proliferation through phosphorylation of *MAPK3*. In addition, increased phosphorylation of *PIK3R1*, *HSP27*, *AKT1*, and *AKT2* is observed in IS tumors. In contrast, IR tumors exhibit higher expression and phosphorylation of *PIK3R1*, potentially leading to activation of the *mTOR* pathway. Meanwhile, patients with the IS subtype tended to have poorer disease-free survival (Fig. 2D).

We focused on protein abundance and performed differential analyses between tumors and NATs (Fig. 2E). Enrichment analysis of DE proteins revealed up-regulation of apoptosis, *ErbB* signaling, and nucleotide excision repair pathways, together with down-regulation of lipolysis in adipocytes, tyrosine metabolism, protein digestion and absorption, and drug metabolism in tumors (Fig. 2F). We next identified proteins with high tumor-specific expression overall and within individual subtypes using differential expression analysis (Fig. 2G and Fig. S1L).

### Genomic landscape across 3 BC subtypes

**Fig. 3.**
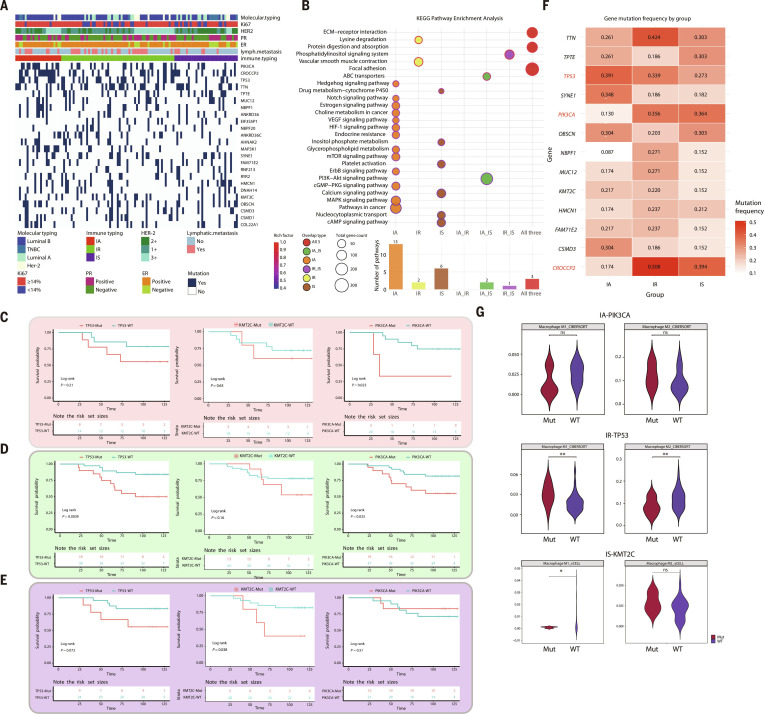
Genomic landscape across three breast cancer subtypes. (A) Genetic-mutation-associated clinicopathologic features of 3 immune clusters. (B) KEGG (Kyoto Encyclopedia of Genes and Genomes) pathways enrichment of mutated gene in the 3 immune clusters. (C to E) Kaplan–Meier curves for overall survival in 3 immune clusters based on the *PIK3CA*/*TP53*/*KMT2C* mutation status (log rank test). (F) Violin diagram shows the infiltration of M1 and M2 macrophages based on the *PIK3CA*/*TP53*/*KMT2C* mutation status. (G) Violin plots comparing the relative abundance of infiltrating M1 and M2 macrophages between mutant (Mut; red) and wild-type (WT; blue) tumor samples. The analysis evaluates mutations in *PIK3CA* (top), *TP53* (middle), and *KMT2C* (bottom), with infiltration levels estimated using CIBERSORT and xCELL algorithms. Statistical significance is denoted as follows: ns, not significant; *P < 0.05; **P < 0.01. *P* values were derived from Wilcoxon test.

The top 10 genes with the highest mutation frequencies among the 3 immune subtypes are shown in the heatmap, including *PIK3CA*, *CROCCP2*, *TP53*, *TTN*, and others (Fig. 3A and Table S6). IA tumors exhibited the highest mutation frequency and tumor mutational burden, consistent with their immune-activated status (Fig. S2A and B). Kyoto Encyclopedia of Genes and Genomes (KEGG) enrichment analysis revealed marked pathway differences among the 3 immune subtypes. In the IA subtype, the most enriched pathways included *VEGF*, *HIF-1*, *MAPK*, *mTOR*, *ErbB*, and Notch signaling, highlighting activation of an angiogenesis-, hypoxia-, and growth-factor-receptor-related network. In contrast, only 2 pathways were significantly enriched in the IR subtype. IS subtype tumors were enriched in 6 pathways, predominantly involving second messenger signaling (*cAMP*/*cGMP*/Ca^2^${}^{+}$) and intracellular transport, consistent with an immunosuppressive or tolerance-like phenotype [17–19]. All 3 subtypes were commonly enriched in extracellular matrix (ECM)–receptor interaction, focal adhesion, and protein digestion and absorption, indicating that matrix- and adhesion-related microenvironmental remodeling is a shared feature across immune states. Among cross-subtype shared pathways, IA and IS jointly enriched **PI3K*–*Akt** signaling and adenosine-triphosphate-binding cassette (ABC) transporters, whereas IR and IS jointly enriched the phosphatidylinositol signaling system (Fig. 3B).

We next assessed the impact of several highly mutated genes on survival within each immune subtype. *PIK3CA*-wild-type tumors in the IA subtype were associated with improved overall survival, whereas IR tumors harboring *TP53* or *PIK3CA* mutations displayed worse survival outcomes. IS tumors carrying *KMT2C* mutations also exhibited poorer clinical outcomes (Fig. 3C to E). These 3 genes (*TP53*, *PIK3CA*, and *KMT2C*) were frequently mutated across all 3 immune subtypes (Fig. 3F). Two independent The Cancer Genome Atlas (TCGA) cohorts showed that *PIK3CA*-wild-type IA-like tumors, *TP53*-wild-type IR-like, and *KMT2C*-wild-type IR-like tumors showed better overall survival than their respective mutant counterparts (Fig. S2C to E). We then compared functional differences between mutant and wild-type tumors for *TP53*, *PIK3CA*, and *KMT2C*. In IA tumors, multiple Hallmark pathways—including transplant rejection, G2/M checkpoint, IFN-α/γ responses, and *MYC* targets v2—were enriched in the *PIK3CA*-mutant group, indicating that *PIK3CA* mutations are associated with cell cycle progression and IFN-related immune activation (Fig. S2F). In IR tumors, *TP53* mutations were linked to diverse functional changes, including activation of inflammatory pathways (inflammatory response, IFN−α, and IFN−γ signaling) and up-regulation of oxidative phosphorylation and glycolysis, suggesting *TP53*-mutation-associated metabolic reprogramming accompanied by enhanced inflammatory signaling (Fig. S2G). The gene set enrichment analysis revealed that epithelial–mesenchymal transition is significantly enriched in *KMT2C*-mutant samples, indicating enhanced mesenchymal and invasive features associated with *KMT2C* alteration. In contrast, multiple metabolic pathways, including fatty acid metabolism, bile acid metabolism, heme metabolism, and oxidative phosphorylation, are preferentially enriched in *KMT2C*-wild-type samples, suggesting preserved metabolic homeostasis in the absence of *KMT2C* mutation. (Fig. S2H). Phosphorylation levels were significantly reduced on inflammation-related proteins but increased on glycolysis-related proteins, whereas lactylation displayed the opposite pattern—lower on glycolysis-related proteins and higher on inflammation-related proteins. In addition, *TP53* and *KMT2C* mutations were significantly correlated with the infiltration of M1 and M2 macrophages in tumors (Fig. 3G).

### Cross-talk between protein phosphorylation and lactylation in BC

**Fig. 4.**
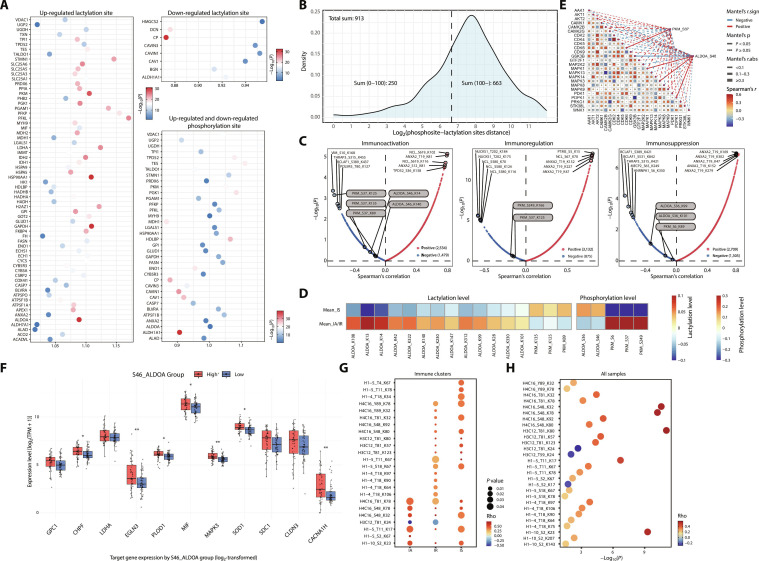
Crosstalk between protein phosphorylation and lactylation in breast cancer. (A) Bubble chart displays fold change in phosphorylation and lactylation modification levels of metabolism related genes, compared between cancer and adjacent tissues. (B) The density curve of phosphosite–lactylation site distances for all phosphorylation and lactylation comodified proteins detected in this study. (C) The correlation volcano map shows the correlation between phosphosites and lactylation sites in 3 immune clusters. (D) The heatmap shows the mean values of the phosphorylation and lactylation modification levels of ALDOA and PKM in immunosuppression (IS) and the other immune clusters. (E) Spearman correlation analysis assesses the relationship between kinase expression levels and the association of kinases with their corresponding phosphorylation sites. (F) Bar plot displaying the expression levels of glycolysis-related genes between the high- and low-abundance groups of ALDOA_S46. *P < 0.05; **P < 0.01; ***P < 0.001; ****P < 0.0001. (G and H) The correlation of phosphorylation and lactylation modification levels among histones.

Lactylation is known to play an important inhibitory role in multiple tumor-metabolism-related signaling pathways [20–24]. Serine/threonine protein kinases exhibit substrate specificity and catalyze phosphorylation in a sequence-dependent manner. Mutual exclusivity between phosphorylation and acetylation on the same protein has been reported and can influence tumor progression [25]. We therefore investigated the cross-talk between phosphorylation and lactylation and asked whether increased lactylation at specific sites might inhibit phosphorylation at neighboring residues. Integrative analysis of phosphosites and lactylation sites identified 913 site pairs, including 250 adjacent pairs separated by fewer than 100 amino acids (Fig. 4A).

We next examined lactylation and phosphorylation levels of several metabolism-related proteins and observed evidence of mutual exclusivity in multiple proteins (*VDAC1*, *PKM*, *ALDOA*, etc.) (Fig. 4B). In particular, lactylation–phosphorylation site pairs in the glycolytic enzymes *ALDOA* and *PKM* (PKM_S37_K125, ALDOA_S46_K14, and ALDOA_S36_K99) were negatively correlated across the 3 immune subtypes (Fig. 4C). In the IS subtype, *PKM* phosphorylation was significantly decreased, whereas *PKM* lactylation was increased, while *ALDOA* phosphorylation was significantly increased and *ALDOA* lactylation decreased (Fig. 4D). Previous studies have shown that phosphorylation at ALDOA_S36 enhances hepatic glucose metabolism through catalytic activation, thereby promoting hepatocellular carcinoma progression [26], whereas lactylation of *ALDOA* reduces enzymatic activity and suppresses glycolysis [27]. Similarly, *PKM* lactylation has been reported to enhance enzymatic activity and increase glycolytic flux, whereas *PKM* phosphorylation suppresses catalytic function and attenuates cellular glycolysis [28,29]. Taken together, these findings led us to hypothesize that elevated *PKM* lactylation and increased *ALDOA* phosphorylation cooperatively enhance glycolysis in IS tumors.

Bioinformatic prediction of kinases potentially targeting PKM_S37, ALDOA_S46, and ALDOA_S36 using the STRING database identified *CDK6* as a candidate regulator of pyruvate kinase M1/2 (PKM) phosphorylation and *MAPK1/3* and *PDPK1* as putative kinases for *ALDOA* (Fig. 4E, Fig. S3A, and Table S7). We stratified BC samples into high- and low-phosphorylation groups based on ALDOA_S46 phosphorylation levels. Differential expression analysis showed that multiple glycolysis-related genes, as well as the putative upstream kinase *MAPK3*, were significantly up-regulated in the high-phosphorylation group (Fig. 4F). Consistently, KEGG enrichment analysis revealed significant enrichment of *MAPK* and **PI3K*–*AKT** signaling pathways (Fig. S3B). We therefore selected key components from these pathways for interaction analysis, which indicated that *PDK1*, *AKT1*, *MYC*, and *MAPK3* all interact with *ALDOA* (Fig. S3C). These predictions suggest that *MAPK3*-driven *ALDOA* phosphorylation may contribute to elevated glycolytic activity in BC. Interestingly, phosphorylation and lactylation of histones were predominantly positively correlated, both within the 3 immune subtypes and in the overall BC cohort (Fig. 4G and H). This pattern suggests that additional regulatory mechanisms govern the interplay between phosphorylation and lactylation on histones.

We observed significant differences in protein expression and lactylation levels across several tumor-metabolism-related signaling pathways in the IS subtype, including lipid and triglyceride metabolism, glycolysis, fatty acid metabolism, oxidative phosphorylation, and hypoxia (Fig. S3D). Lipid and triglyceride metabolism, glycolysis, and hypoxia pathways were significantly up-regulated in the IS subtype compared with IA and IR. Low lactylation in lipid and triglyceride metabolism and high lactylation in glycolysis and hypoxia suggest that elevated lactylation may contribute to activation of glycolysis and hypoxia pathways in IS tumors.

Recent studies have shown that lipid- and lactate-enriched TMEs reduce the cytotoxicity of effector T cells [10,30,31], due to the accumulation of lipid droplets and decreased extracellular pH [32,33]. Therefore, we examined whether lipid-associated lactylation sites were down-regulated and glycolysis-associated lactylation sites were up-regulated in IS tumors (Fig. S3E). We observed a significant positive correlation between lipid lactylation levels and the protein levels of IFN−γ pathway-related effectors (Fig. S3F) and a negative correlation between glycolysis lactylation sites and IFN−γ pathway-related effectors (Fig. S3G). These findings suggest that low-level lactylation of lipid metabolism-related genes and high-level lactylation of glycolysis-related genes may promote the formation of an immunosuppressive microenvironment in IS tumors.

### Alterations in histone lactylation across 3 subtypes

**Fig. 5.**
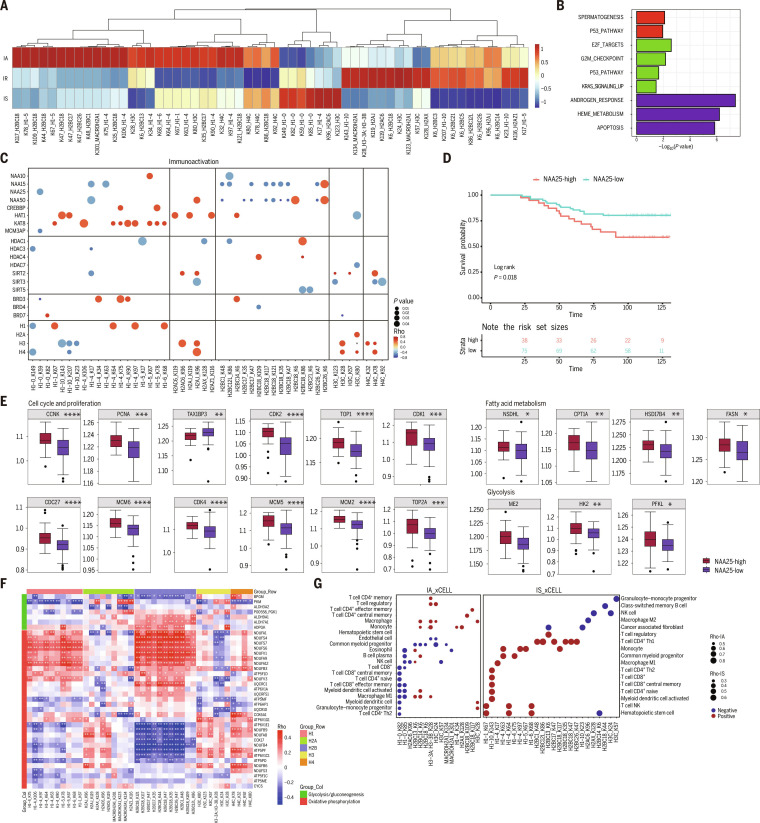
Alterations in histone lactylation across 3 subtypes. (A) Heatmap displaying the mean levels of all histone lactylation modifications in 3 immune clusters. (B) KEGG (Kyoto Encyclopedia of Genes and Genomes) enrichment analysis based on the histone with high level lactylation modifications of immunoactivation (IA), immunoregulation (IR), and immunosuppression (IS) immune subtypes, respectively. (C) Significant associations between core histone lactylation sites and acyltransferase, deacylases, and bromodomain-containing proteins in IA tumors. (D) Kaplan–Meier analysis of overall survival according to *NAA25* protein expression. (E) The genes related to proliferation and metabolism protein expression between NAA25-high and NAA25-low. (F) Heatmap displays the correlation between proteins related to glycolysis, oxidative phosphorylation pathways, and histone lactylation modification. (G) Spearman correlation between the histone lactate modification immune cell infiltration abundance estimated from xCell.

Histone lactylation is a recently described PTMs that occurs on lysine residues of histones and modulates transcriptional regulation [34]. It has also been implicated in diverse biological processes, including tumor-associated metabolic reprogramming and immune responses, embryonic development, inflammatory cascades, and the pathogenesis of neuropsychiatric disorders [35–37]. To characterize histone lactylation across the 3 immune subtypes, we identified histone lactylation sites that differed significantly among IA, IR, and IS tumors. IA and IS exhibited broadly distributed histone lactylation, whereas IR displayed a distinct pattern dominated by lactylation of the histone H1.0 variant (Fig. 5A and Table S8). Enrichment analysis of subtype-specific lactylation gains revealed that the p53 pathway was enriched in both IA and IR, G2/M checkpoint and KRAS signaling were characteristic of IR, and apoptosis-related pathways were enriched in IS (Fig. 5B).

Next, we examined associations between histone lactylation levels and key chromatin regulators, including histone acetyltransferases, histone deacetylases (HDACs), and bromodomain proteins (BRDs) (Fig. 5C and Fig. S4A and B). From IA to IS tumors, as immune suppression increased, positive correlations between histone lactylation and these regulators became progressively stronger. In contrast, *NAA15* and *NAA25* consistently showed negative correlations with most regulators across all 3 subtypes. Consistent with this pattern, low expression of *NAA15* or *NAA25* was associated with improved overall survival (Fig. 5D and Fig. S4C). In the *NAA25*-low cohort, genes involved in cell cycle progression and proliferation, fatty acid metabolism, and glycolysis were significantly down-regulated, and a similar trend was observed in the *NAA15*-low group (Fig. 5E and Fig. S4D). The independent TCGA cohort showed that *NAA15* or *NAA25* low expression tumors showed better overall survival than high expression tumors (Fig. S4E and F).

Building on the mechanistic link between glucose metabolism and lactylation dynamics, we performed systematic correlation analyses of histone lactylation with key regulators of glycolysis and oxidative phosphorylation (Table S9). Multiple ubiquinone oxidoreductase subunits displayed strong positive correlations with H1 and H2B lactylation, whereas a gene cluster (*ATP6V1E1*, *ATP5PF*, *ATP6V1C1*, *ATP5PD*, *NDUFA8*, *NDUFB4*, *NDUFB9*, and *COX17*) exhibited inverse associations with H1 and H2B modifications. In addition, glycolysis-related genes (*ALDH7A1*, *ALDH9A1*, and *ADPGK*) were positively correlated with H2B lactylation (Fig. 5F). Pan-immune cell infiltration was significantly negatively correlated with H1 lactylation in IA tumors, whereas IS tumors showed strong positive correlations (Fig. 5G and Table S10). To validate these observations, we stratified BC samples into high- and low-H1 lactylation groups, and the top 30 up-regulated genes in the high-lactylation group were defined as an H1 lactylation signature (Fig. S4G and H). This signature was then correlated with immune cell infiltration in the PDC000173 cohort, yielding results consistent with those in Fig. 5G (Fig. S4I). Together, these findings indicate that the relationship between histone lactylation and immune cell infiltration is highly context dependent and tightly coupled to the immune state of the TME.

### Tumor–NAT differential proteomic and PTM signatures identify candidate treatment targets

According to the previous proteomic analysis of differences between cancer and adjacent noncancerous tissues (Fig. 2E to G and Fig. S1G), IA tumors showed strong molecular links to the **JAK*–*STAT** signaling pathway, as evidenced by significant overexpression of *PTPN2* and *STAT1* in the IA subtype [38,39]. *NAT10*, *CD3E*, *IL4I1*, and *CD40* were further highlighted as candidate biomarkers for IA immunotherapy based on their established roles in regulating tumor immune infiltration [40–42]. For IR tumors, *SEC13*, *FLII*, *DNAJC13*, *FBXO22*, and *BAG5* emerged as potential biomarkers from integrative molecular profiling. Mechanistically, *SEC13* and *BAG5* participate in regulation of the *mTOR* pathway, with *SEC13* displaying oncogenic potential through promotion of BC progression [43–45]. The selected IR biomarkers also exhibited distinct immunomodulatory properties: *FLII* and *FBXO22* were negatively correlated with *PD-L1* expression, suggesting roles in antitumor immune evasion [46,47], whereas *DNAJC13* contributes to adaptive immunity through T-cell-receptor-mediated antigen recognition and Toll-like-receptor-dependent activation of cytotoxic T cell responses [48]. In IS tumors, TME analyses identified *VCAN* and *PDIA5* as key mediators of immunosuppressive niche formation and resistance to immunotherapy [49–51]. Elevated *RBCK1* expression had dual clinical implications: It was associated with immunosuppression and reduced immunotherapy efficacy yet simultaneously increased tumor sensitivity to antiangiogenic therapies [52]. *AGK* promoted angiogenesis via activation of the NF−κB pathway. *GREM1* functioned as a multifaceted regulator in BC pathogenesis [53,54], enhancing tumor proliferation and driving metastatic dissemination [55,56]. Receiver operating characteristic (ROC) analysis showed that each subtype-enriched gene could accurately discriminate its corresponding tumor subtype (Fig. S5A). We validated these subtype markers in the PDC000173 cohort, where they also demonstrated robust predictive performance for immune subtype classification (Fig. S5B).

**Fig. 6.**
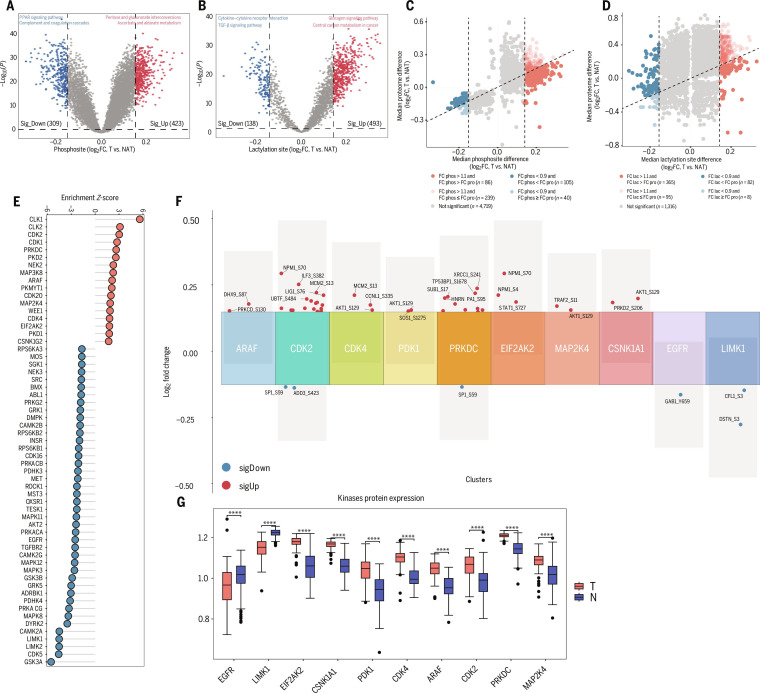
Tumor-NAT differential proteomic and posttranslational modification (PTM) signatures identify candidate treatment targets. (A and B) Volcano plot depicting differentially expressed phosphosites (A) and lactylation sites (B) between tumors and paired NATs (*t* test). Representative pathways are annotated. TGF-β, transforming growth factor-β. (C and D) Scatter plot depicting comparison of abundance changes between phosphosites or lactylation sites and their corresponding proteins (*t* test). (E) Significant (false discovery rate < 0.05) kinase signatures between tumors and paired NATs assessed by PTM signature enrichment analysis (PTM-SEA). (F) Diagram showing the median log_2_ fold change (log_2_FC) of kinase substrates phosphorylation. Pink and blue colors indicate up-regulated or down-regulated phosphosites between tumors and paired NATs, respectively. (G) The expression of phosphorylation kinase in tumor and normal tissue. ****P < 0.0001.

We simultaneously focused on the differences in phosphorylation and lactylation levels between cancerous tissues and adjacent noncancerous tissues and performed differential analyses between tumors and NATs (Fig. 6A and B). We further identified 325 phosphosites with more than 1.1-fold up-regulation in tumors compared with NATs, of which 86 displayed larger changes in phosphosite abundance than in the corresponding protein abundance (Fig. 6C). For lactylation, 460 sites were up-regulated by at least 1.1-fold, and 365 of these exhibited greater changes than their matched protein abundance (Fig. 6D). We next inferred kinase activity from substrate phosphorylation levels and the phosphorylation status of kinase-activating sites. Phosphosite-specific signature enrichment analysis (PTM-SEA) [57] identified 59 kinases, 17 of which showed increased activity in BC (Fig. 6E). Among these, *CDK2* and *CDK4* are well-established regulators of the tumor cell cycle. We found that *CDK2* and *CDK4* were associated with elevated phosphorylation of substrates involved in cell cycle arrest and DNA damage responses, including *LIG1*, *SAMHD1*, *TERF2*, *CCNL1*, and *CCNT2* (Fig. 6F and G). These proteomic and phosphoproteomic findings highlight candidate therapeutic targets for BC.

### Key oncogenic pathways and therapeutic opportunities for 3 immune subtypes

**Fig. 7.**
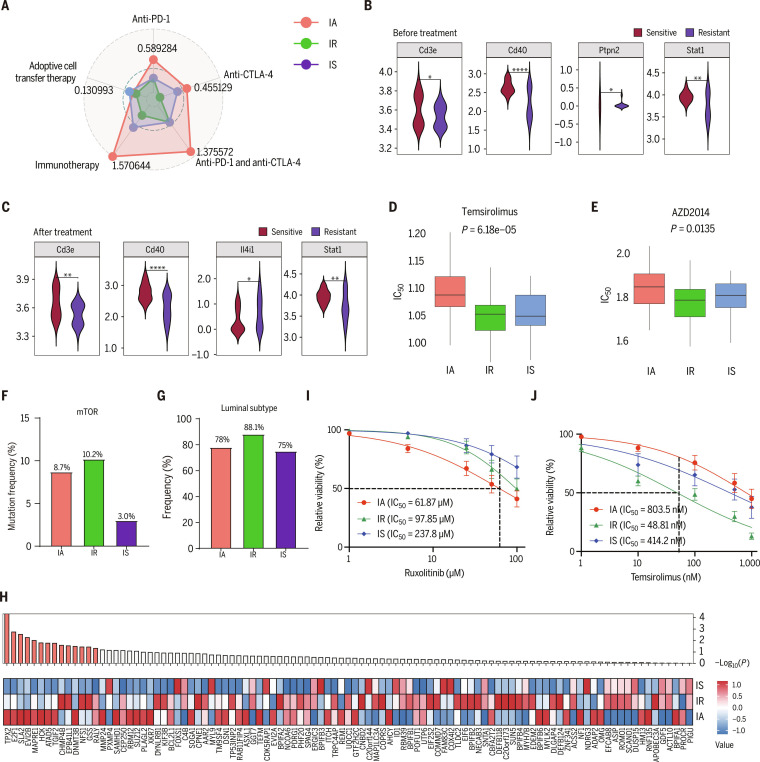
Key oncogenic pathways and therapeutic opportunities for 3 immune subtypes. (A)The radar chart displays 3 immune typing immune therapy sensitivity scores, based on the TIGER (Tumor Immunotherapy Gene Expression Resource) database. CTLA, cytotoxic T lymphocyte-associated antigen-4. (B and C) Differential expression of immunoactivation (IA) subtype signature genes between immunotherapy-sensitive and -resistant groups in the GSE124821 dataset. *P < 0.05; **P < 0.01;****P < 0.0001. (D and E) Drug sensitivity analysis of temsirolimus (D) and AZD2014 (E) across different immune subtypes (IA, immunoregulation [IR], and immunosuppression [IS]). Box plots show the median inhibitory concentration (IC_50_) values for the 3 subtypes. (F) The mutational frequency of mTOR in 3 immune clusters. (G) The proportion of luminal subtypes in the 3 immune clusters. (H) Obtaining antiangiogenic-therapy-related resistance, related genes were identified through the Genomics of Drug Sensitivity in Cancer (GDSC) database; the protein expression between IA, IR, and IS immune subtypes was calculated using ANOVA test, and a mean heatmap was plotted. (I and J) Drug sensitivity assays for the IA and IR subtypes of the PDO (patient-derived organoid) models. Data are presented as means ± SEM, with IC_50_ values indicated in the legend.

Based on these multiomics subtype characteristics, we next explored subtype-specific therapeutic vulnerabilities in BC. The IA subtype was associated with an immune-activated phenotype and activation of *JAK/STAT* signaling. Using the TIGER (Tumor Immunotherapy Gene Expression Resource) database, we found that the IA subtype had the highest predicted immunotherapy response score (Fig. 7A and Table S11). Consistently, IA tumors displayed high expression of immune checkpoint molecules, including *LAG3*, *CTLA-4*, and *PD-1* (*PDCD1*) (Fig. S5C). Moreover, immunotherapy data from Hollern et al. showed that multiple IA subtype markers were elevated in the immunotherapy-sensitive group (Fig. 7B and C). Together, these findings suggest that IA-subtype BCs are more likely to benefit from immunotherapy. In addition to highly activated *mTOR* signaling, IR tumors exhibited the highest *mTOR* mutation frequency and the largest proportion of luminal BCs, suggesting that this subtype may derive particular benefit from *mTOR* inhibitors (Fig. 7D and E). Drug sensitivity prediction indicated that the 2 *mTOR* inhibitors temsirolimus showed the greatest predicted sensitivity in IR tumors (Fig. 7F and G). Given the highly activated *VEGF* signaling pathway and elevated *RBCK1* expression in IS tumors, these cancers may be particularly responsive to antiangiogenic therapy. Analysis of a gene set associated with resistance to antiangiogenic therapy showed that 14 genes differed significantly among the 3 subtypes and were expressed at lower levels in IS tumors (Fig. 7H). This pattern supports our hypothesis and suggests that patients with IS subtype tumors may benefit most from antiangiogenic therapy. We collected human BC tissue and established patient-derived organoid (PDO) models for drug sensitivity testing. In parallel, we performed transcriptome sequencing on the BC tissue to distinguish the 3 subtypes. The results showed that the IA subtype had the lowest median inhibitory concentration (IC_50_) for *JAK* inhibitors, the IR subtype had the lowest IC_50_ for *mTOR* inhibitors, and the IS subtype had the lowest IC_50_ for antiangiogenic drugs (Fig. 7I and J).

## Discussion and Conclusion

BC multiomics studies, particularly those dissecting the immune microenvironment, have been constrained by limited omics depth, modest cohort size, and incomplete clinical translation. In this work, we used an integrated proteogenomic, phosphoproteomic, and lactylomic framework to define BC immune subgroups and construct an immune–metabolic atlas that links genomic alterations and PTM-governed signaling to tumor immune states. This layered view reveals how phosphorylation and lactylation jointly modulate glycolytic flux, metabolic plasticity, and immune activity, providing mechanistic insight into how tumors achieve and maintain immune escape.

Our integration of phosphoproteomic and lactylomic datasets further uncovers a layer of nonhistone PTM cross-talk that fine-tunes metabolic output in an immune context-dependent manner. The reciprocal relationship between *ALDOA* and *PKM* phosphorylation and lactylation (Fig. 4C and D), together with their subtype-specific PTM patterns, suggests a PTM-based “metabolic switch” in which phosphorylation and lactylation redistribute glycolytic flux to support distinct immune states. Kinase–substrate prediction using STRING pinpointed *CDK6* as a candidate kinase for PKM_S37 and *MAPK1/3* and *PDPK1* as putative kinases for *ALDOA* (Fig. 4E and Fig. S3A), while ALDOA_S46-high tumors showed coordinated up-regulation of glycolysis-related genes and *MAPK3* (Fig. 4F and Fig. S3B and C). These findings imply that kinase-driven control of *ALDOA* and *PKM* phosphorylation interfaces with lactylation dynamics to reshape glycolysis in different immune subtypes, extending the classical Warburg paradigm toward a PTM-centric model of metabolic plasticity. This concept aligns with recent work from Tongji University showing that *RCC2* lactylation promotes mitotic arrest deficient 2 like 1 (MAD2L1)-mediated mitotic progression under high-glucose conditions, underscoring the dual role of PTMs in orchestrating tumor metabolism and immune escape [58].

Histone lactylation adds an additional epigenetic layer to this regulatory network. We found that subtype-specific patterns of H1 and H2B lactylation were tightly coupled to oxidative phosphorylation and glycolytic programs (Fig. 5F and Table S9) and that pan-immune cell infiltration was negatively correlated with H1 lactylation in IA tumors but positively correlated in IS tumors (Fig. 5G, Fig. S4G, and Table S10). These context-dependent associations position histone lactylation as a tunable epigenetic switch that can either restrict or permit immune infiltration depending on the immune state of the TME, echoing data from the Moffitt Cancer Center showing that *PERK-*driven glucose metabolism enhances the immunosuppressive activity of monocyte-derived macrophages via histone lactylation [59]. Moreover, *NAA15* and *NAA25* showed consistent negative correlations with histone-lactylation-linked regulatory programs, and low *NAA15/NAA25* expression was associated with down-regulation of proliferation, fatty acid metabolism, and glycolysis and with improved overall survival (Fig. 5D and E and Fig. S4C and D). These observations implicate the *N*-acetyltransferase A (NatA) complex as a candidate metabolic–immune rheostat, conceptually parallel to emerging evidence that microbial metabolites modulate tumor immune suppression through histone modifications [60].

Our multiomics framework also refines the therapeutic landscape for each immune subtype. In IA tumors, *JAK/STAT* hyperactivation (Fig. 7A), together with elevated *PD-1* and *LAG3* expression (Fig. S1A), is concordant with the highest predicted immunotherapy response scores derived from the TIGER resource (Fig. 7B) and up-regulation of IA markers in an ICI-sensitive cohort (Fig. S6D and E) [56,57].

Preclinical and early-phase clinical studies have shown that *JAK* inhibition with ruxolitinib can enhance T and NK cell function and improve tumor control when combined with *PD-1* blockade [61] and that adding the *JAK1* inhibitor itacitinib to pembrolizumab can modulate treatment responses in metastatic non-small-cell lung cancer [62]. Extrapolating from these findings, IA tumors—characterized by robust *JAK/STAT* signaling and a preexisting inflamed microenvironment—may be particularly amenable to combinatorial strategies integrating ICIs with *JAK* pathway modulation.

In contrast, IR tumors are defined by *mTOR* pathway activation, increased *mTOR* mutation frequency, and a predominance of luminal subtypes (Fig. 7A and Fig. S6F and G), together with elevated *CDK2/CDK4* activity toward substrates involved in cell cycle and DNA damage responses (Fig. 6I and J and Fig. S5G). Drug sensitivity modeling indicated that the 2 *mTOR* inhibitors temsirolimus exhibited the strongest predicted efficacy in IR tumors (Fig. 7C and D), suggesting that IR-subtype BCs may benefit preferentially from *mTOR*-targeted regimens.

IS tumors, by contrast, exhibited activation of **VEGF/RAF1*–*MAPK** signaling and high *RBCK1* expression (Fig. 7A and Fig. S6C), along with a favorable expression profile of genes associated with sensitivity to antiangiogenic therapy (Fig. 7E). These features nominate IS tumors as promising candidates for antiangiogenic strategies, possibly combined with metabolic modulators aimed at reversing lactate- and lipid-enriched immunosuppressive niches.

Several limitations should be acknowledged. First, our cohort size, although substantial for deep proteogenomic profiling, remains modest and is restricted to patients from a single center and ethnic background, which may limit generalizability. Second, the multiomics analyses are largely correlative; mechanistic validation of key PTM interactions (e.g., *ALDOA/PKM* phosphorylation–lactylation switches and NatA-mediated regulation of histone lactylation) will require targeted perturbation experiments in vitro and in vivo. Third, treatment response predictions (immunotherapy, *mTOR* inhibition, and antiangiogenic therapy) are inferred from omics signatures and external datasets rather than prospective clinical trials in these subtypes. Future investigations using larger, more balanced, and independent cohorts will be necessary to confirm the robustness and broader applicability of the proposed immune classification.

## Materials and Methods

### Immune infiltration and functional enrichment analysis

To estimate the abundance of immune cell populations and functional features, GSVA was performed on the proteomic profiles of tumor samples. We utilized the C7 (immunologic signature gene sets) from the MSigDB (Molecular Signature Database) database (via the msigdbr R package, v7.5.1). To ensure biological relevance, we specifically curated gene sets corresponding to distinct immune cell lineages. These gene sets were further aggregated into 28 representative immune cell types by calculating the median GSVA score within each category to represent the overall variation of that specific immune population. The GSVA was implemented using the OmicStudio platform (https://www.omicstudio.cn/tool) with the following parameters: method = “gsva”, kcdf = “Gaussian”, tau = 1, and gene set size constraints of 10 to 500 genes. For cross-platform validation, bulk RNA-seq data were analyzed using the TIMER 2.0 tool (https://compbio.cn/timer2/), specifically selecting the BC model. Multiple deconvolution algorithms, including TIMER, CIBERSORT, xCell, and MCP-counter, were used to provide comprehensive estimations of immune infiltration.

Identification of immune subtype unsupervised hierarchical clustering was performed on the basis of the relative abundance of the 28 immune cell types using the pheatmap R package (v1.0.12). The clustering was executed with Euclidean distance and the “complete” linkage method, followed by *Z*-score normalization across samples. Based on the distinct immune landscapes, tumors were categorized into 3 subtypes: IA, characterized by high infiltration of proinflammatory cells and low abundance of anti-inflammatory cells; IS, characterized by low overall immune cell abundance; IR, characterized by an intermediate state of various immune cell populations. Visualization of the clustering results and subsequent analyses was generated using the ggplot2 R package (v3.4.0). Transcriptomic profiling of PDOs was performed, and the resulting data were categorized into the aforementioned immune subtypes using the same bioinformatic pipeline and parameters.

### Enrichment analysis

DE genes (DEGs) were identified using DESeq2 (RNA-seq) or *t* test (other omics). We selected the genes, which had *P* < 0.05 and absolute fold change larger than 2 (transcriptomics) or 1.1 (proteomics, phosphoproteomics, and lactylomics). KEGG enrichment analysis was conducted on the basis of DEGs between cancer and adjacent tissues. To designate the representative pathways of immune subtypes, we selected DEGs using a “one versus rest” approach and then underwent gene set enrichment analysis of C2-CP: canonical pathways gene set and Hallmark gene set in MSigDB database (msigdbr package: 7.5.1) to calculate the normalized enrichment score (NES) for each gene set in each sample.

### Kinase prediction

To predict ALDOA-S46-, ALDOA-S36-, and PKM-S37-related kinases, GPS5.0 was used (https://gps.biocuckoo.cn/index.php) [63]. We entered protein sequence(s) in FASTA format and set the threshold to high. According to score and cutoff, correlation is calculated. We evaluated site-specific phosphorylation pathway enrichment using KSEA (kinase–substrate enrichment analysis) app (https://casecpb.shinyapps.io/ksea/) [64] and inferred relative kinase activity based on the log fold change values of phosphorylation modification sites between cancer and adjacent tissues. Database includes PhosphatSitePlus and NetworKIN. NetworKIN score cutoff is 2. The *P* value cutoff is <0.05.

### Survival analysis

Based on the patient’s disease-free survival or overall survival, survival status, we performed a 3 subtypes survival analysis, created a survival object using the Surv function, and fit a survival curve model using the Survfit function. We used the Survdiff function to perform log rank tests and the ggsurvplot function to plot survival curves. We also used using R packages: Survival 3.2.13 and Surviviner 0.4.6.

### ROC analysis

ROC analysis was used to evaluate the ability of each subtype of protein as a biomarker to distinguish between cancer tissue and normal tissue adjacent to cancer. In addition, we used logic to fit different proteins to evaluate the impact of multiple independent variables (protein abundance) on the dependent variable (whether it is cancer). We used R package pROC 1.18.5 and ggplot2 3.4.0 for plotting.

### Sample collection and ethics

A prospective cohort comprising 115 BC tumor specimens and 135 matched NAT samples was collected from Harbin Medical University Cancer Hospital for multiomics profiling. All participants provided written informed consent, and the study protocol was approved by the Institutional Review Board of Harbin Medical University Cancer Hospital, conforming to the Declaration of Helsinki. Clinicopathological parameters—including age, tumor dimensions, histologic subtype/grade, nodal status, and estrogen receptor/progesterone receptor/HER2/Ki67 expression—were documented (Table S1). Overall survival was calculated from surgery to death.

### Nucleic acid coextraction and sequencing

WES and RNA-seq were performed by BGI Genomics (China). DNA and RNA were coextracted from frozen tissues using the AllPrep DNA/RNA Kit (QIAGEN, catalog no. 80224) per the manufacturer’s protocols.

### WES library construction and analysis

Libraries were prepared with the SureSelect Human All Exon v6 capture system (Agilent) and sequenced on a DNB-seq platform (BGI Genomics). Genomic alterations—including single-nucleotide variants, short/long insertions–deletions, and copy number alterations—were systematically characterized.

### RNA-seq processing

Paired-end sequencing (150 bp) was conducted on the DNB-seq platform, achieving a 97.94% average genomic alignment rate. Quality-filtered reads were aligned to reference transcripts via Bowtie2 (v2.3.4.3). Gene expression was quantified as FPKM (fragments per kilobase per million mapped reads).

### Protein extraction and digestion

#### Protein isolation

Tissues were pulverized in liquid nitrogen, washed with phosphate-buffered saline, and precipitated in 10% trichloroacetic acid/acetone (4× volume, −20 °C, 5 h). Pellets were centrifuged (6,500*g*, 5 min), washed thrice with chilled acetone, and solubilized in lysis buffer (1% SDS, protease/phosphatase inhibitors, 3 μM trichostatin A, and 50 mM nicotinamide). Lysates were sonicated (Scientz ultrasonic processor, ice, and intermittent cycles), clarified by centrifugation (12,000*g*, 10 min, 4 °C), and quantified via bicinchoninic acid assay.

#### Trypsin digestion

Protein aliquots were acetone-precipitated, reconstituted in 200 mM triethylammonium bicarbonate, and digested with trypsin (1:50, w/w, 37°C, overnight). Peptides were reduced (5 mM dithiothreitol, 56°C, 30 min), alkylated (11 mM indole-3-acetic acid, room temperature, 15 min, dark), desalted (C18 SPE), and lyophilized.

### PTM enrichment

#### Phosphopeptides

Peptides were incubated with immobilized metal affinity chromatography microspheres in loading buffer (50% acetonitrile [ACN]/0.5% acetic acid). After washing (50% ACN/0.5% acetic acid → 30% ACN/0.1% trifluoroacetic acid), phosphopeptides were eluted with 10% NH_4_OH and lyophilized.

#### Lysine-lactylated peptides

Peptides were incubated with anti-K^lac^ antibody beads (PTM-1404, PTM Bio) in enrichment buffer [100 mM NaCl, 1 mM EDTA, 50 mM tris-HCl, and 0.5% NP-40 (pH 8.0); 4 °C, overnight). Beads were washed (enrichment buffer ×4 → H_2_O ×2), and lactylated peptides were eluted (0.1% trifluoroacetic acid ×3), desalted (C18 ZipTip), and dried.

### Liquid chromatography–tandem mass spectrometry configuration

#### Chromatography

All analyses used identical reversed-phase columns (25 cm × 100 μm, self-packed) with solvent A: 0.1% formic acid and 2% ceric ammonium nitrate; solvent B: 0.1% formic acid in ceric ammonium nitrate; and flow rate: 450 nl/min (nanoElute UHPLC, Bruker Daltonics). Gradient Profiles are as follows:

**Table:** 

Analysis type	Gradient program
Global proteome	6% to 24% B (70 min), 24% to 35% B (14 min),35% to 80% B (3 min), 80% B (3 min)
Phosphoproteome	2% to 22% B (76 min), 22% to 35% B (6 min), 35% to 90% B (4 min), 90% B (4 min)
Lactylome	7% to 24% B (40 min), 24% to 32% B (12 min), 32% to 80% B (4 min), 80% B (4 min)

### Mass spectrometry

Instrument is timsTOF Pro (Bruker Daltonics) with nanoelectrospray ionization. Tandem mass spectrometry range is a mass/charge ratio of 100 to 1,700. Fragmentation mode is parallel accumulation serial fragmentation (PRM-PASEF for phosphoproteomics). Precursor charge states are 0 to 5; dynamic exclusion is 30 s (proteome) and 24 s (PTMs); electrospray ionization voltage is 1.75 kV (proteome), 1.70 kV (phosphoproteome), and 1.60 kV (lactylome).

### Database searching

Raw spectra were searched against the Swiss-Prot human database (20,389 entries; 2023_01) with reverse decoys using MaxQuant. Parameters are as follows: enzyme: trypsin/P (max 2 missed cleavages for proteome/phosphoproteome; max 4 for lactylome); mass tolerances: ±20 parts per million (precursor/fragment); fixed modification: carbamidomethylation (C); variable modifications: N-terminal acetylation and methionine oxidation; PTM-specific variables: phosphorylation (S/T/Y) for phosphoproteome and Lys lactylation for lactylome; false discovery rate: ≤1% at peptide/protein levels (minimum 1 unique peptide).

### Drug sensitivity prediction

Drug sensitivity analysis was performed using the GDSC (Genomics of Drug Sensitivity in Cancer) database, which provided the Cell_line_RMA_proc_basalExp dataset along with BC drug sensitivity data (BRCA_IC_Sun Nov 17 04_36_20 2024). Gene-specific drug sensitivity analysis was conducted on the basis of proteomic data. The analysis utilized the oncoPredict v1.2 package for drug sensitivity prediction, and statistical significance was calculated using analysis of variance (ANOVA) to assess the differences in drug sensitivity across the immune subtypes (IA, IR, and IS). The differential analysis and plotting were performed using the ggpubr 0.6.0 and ggplot2 3.5.1 packages. The calcPhenotype function was applied with the parameters: batchCorrect = “eb” and minNumSamples = 10 to ensure accurate results.

## Data Availability

The datasets utilized in this research are accessible through correspondence with the primary investigator following a reasonable request.

## References

[1] Siegel RL, Miller KD, Wagle NS, Jemal A. Cancer statistics, 2023. CA Cancer J Clin. 2023;73(1):17–48. 36633525 10.3322/caac.21763

[2] Adams S, Schmid P, Rugo HS, Winer EP, Loirat D, Awada A, Cescon DW, Iwata H, Campone M, Nanda R, et al. Pembrolizumab monotherapy for previously treated metastatic triple-negative breast cancer: Cohort A of the phase II KEYNOTE-086 study. Ann Oncol. 2019;30(3):397–404. 30475950 10.1093/annonc/mdy517

[3] Emens LA, Molinero L, Loi S, Rugo HS, Schneeweiss A, Dieras V, Iwata H, Barrios CH, Nechaeva M, Nguyen-Duc A, et al. Atezolizumab and nab-paclitaxel in advanced triple-negative breast cancer: Biomarker evaluation of the IMpassion130 study. J Natl Cancer Inst. 2021;113(8):1005–1016. 33523233 10.1093/jnci/djab004 PMC8328980

[4] Lin R, Wang Y, Lu Q, Tang B, Li J, Gao H, Gao Y, Li H, Ding C, Wen J, et al. All-perovskite tandem solar cells with 3D/3D bilayer perovskite heterojunction. Nature. 2023;620(7976):994–1000. 37290482 10.1038/s41586-023-06278-z

[5] Veglia F, Sanseviero E, Gabrilovich DI. Myeloid-derived suppressor cells in the era of increasing myeloid cell diversity. Nat Rev Immunol. 2021;21(8):485–498. 33526920 10.1038/s41577-020-00490-y PMC7849958

[6] Zhang K, Hocker JD, Miller M, Hou X, Chiou J, Poirion OB, Qiu Y, Li YE, Gaulton KJ, Wang A, et al. A single-cell atlas of chromatin accessibility in the human genome. Cell. 2021;184(24):5985–6001 e19. 34774128 10.1016/j.cell.2021.10.024 PMC8664161

[7] Cortes J, Cescon DW, Rugo HS, Nowecki Z, Im SA, Yusof MM, Gallardo C, Lipatov O, Barrios CH, Holgado E, et al. Pembrolizumab plus chemotherapy versus placebo plus chemotherapy for previously untreated locally recurrent inoperable or metastatic triple-negative breast cancer (KEYNOTE-355): A randomised, placebo-controlled, double-blind, phase 3 clinical trial. Lancet. 2020;396(10265):1817–1828. 33278935 10.1016/S0140-6736(20)32531-9

[8] Schmid P, Rugo HS, Adams S, Schneeweiss A, Barrios CH, Iwata H, Dieras V, Henschel V, Molinero L, Chui SY, et al. Atezolizumab plus nab-paclitaxel as first-line treatment for unresectable, locally advanced or metastatic triple-negative breast cancer (IMpassion130): Updated efficacy results from a randomised, double-blind, placebo-controlled, phase 3 trial. Lancet Oncol. 2020;21(1):44–59. 31786121 10.1016/S1470-2045(19)30689-8

[9] Keren L, Bosse M, Marquez D, Angoshtari R, Jain S, Varma S, Yang SR, Kurian A, Van Valen D, West R, et al. A structured tumor-immune microenvironment in triple negative breast cancer revealed by multiplexed ion beam imaging. Cell. 2018;174(6):1373–1387 e19. 30193111 10.1016/j.cell.2018.08.039 PMC6132072

[10] Watson MJ, Vignali PDA, Mullett SJ, Overacre-Delgoffe AE, Peralta RM, Grebinoski S, Menk AV, Rittenhouse NL, DePeaux K, Whetstone RD, et al. Metabolic support of tumour-infiltrating regulatory T cells by lactic acid. Nature. 2021;591(7851):645–651. 33589820 10.1038/s41586-020-03045-2 PMC7990682

[11] Hoxhaj G, Manning BD. The PI3K-AKT network at the interface of oncogenic signalling and cancer metabolism. Nat Rev Cancer. 2020;20(2):74–88. 31686003 10.1038/s41568-019-0216-7 PMC7314312

[12] Zhang D, Qiu D, Gibson MA, Zheng Y, Fraser HL, StJohn DH, Easton MA. Additive manufacturing of ultrafine-grained high-strength titanium alloys. Nature. 2019;576(7785):91–95. 31802014 10.1038/s41586-019-1783-1

[13] Mendiratta G, Ke E, Aziz M, Liarakos D, Tong M, Stites EC. Cancer gene mutation frequencies for the U.S. population. Nat Commun. 2021;12(1):5961. 34645806 10.1038/s41467-021-26213-y PMC8514428

[14] Liu J, Liu W, Wan Y, Mao W. Crosstalk between exercise and immunotherapy: Current understanding and future directions. Research. 2024;7:0360. 38665847 10.34133/research.0360 PMC11045263

[15] Qi F, Li J, Qi Z, Zhang J, Zhou B, Yang B, Qin W, Cui W, Xia J. Comprehensive metabolic profiling and genome-wide analysis reveal therapeutic modalities for hepatocellular carcinoma. Research. 2023;6:0036. 37040510 10.34133/research.0036 PMC10076022

[16] Sun S, Ma J, Zuo T, Shi J, Sun L, Meng C, Shu W, Yang Z, Yao H, Zhang Z. Inhibition of PCSK9: A promising enhancer for anti-PD-1/PD-L1 immunotherapy. Research. 2024;7:0488. 39324018 10.34133/research.0488 PMC11423609

[17] Cui C, Merritt R, Fu L, Pan Z. Targeting calcium signaling in cancer therapy. Acta Pharm Sin B. 2017;7(1):3–17. 28119804 10.1016/j.apsb.2016.11.001 PMC5237760

[18] Jennings MR, Munn D, Blazeck J. Immunosuppressive metabolites in tumoral immune evasion: Redundancies, clinical efforts, and pathways forward. J Immunother Cancer. 2021;9(10). 10.1136/jitc-2021-003013 PMC8527165 34667078

[19] Zhang H, Liu Y, Liu J, Chen J, Wang J, Hua H, Jiang Y. cAMP-PKA/EPAC signaling and cancer: The interplay in tumor microenvironment. J Hematol Oncol. 2024;17(1):5. 38233872 10.1186/s13045-024-01524-x PMC10792844

[20] Zhao Q, Wang Q, Yao Q, Yang Z, Li W, Cheng X, Wen Y, Chen R, Xu J, Wang X, et al. Nonenzymatic lysine D-lactylation induced by glyoxalase II substrate SLG dampens inflammatory immune responses. Cell Res. 2025;35(2):97–116. 39757301 10.1038/s41422-024-01060-w PMC11770101

[21] Hong H, Han H, Wang L, Cao W, Hu M, Li J, Wang J, Yang Y, Xu X, Li G, et al. ABCF1-K430-lactylation promotes HCC malignant progression via transcriptional activation of HIF1 signaling pathway. Cell Death Differ. 2025;32(4):613–631. 39753865 10.1038/s41418-024-01436-w PMC11982231

[22] Yang Z, Yan C, Ma J, Peng P, Ren X, Cai S, Shen X, Wu Y, Zhang S, Wang X, et al. Lactylome analysis suggests lactylation-dependent mechanisms of metabolic adaptation in hepatocellular carcinoma. Nat Metab. 2023;5(1):61–79. 36593272 10.1038/s42255-022-00710-w

[23] Mao Y, Zhang J, Zhou Q, He X, Zheng Z, Wei Y, Zhou K, Lin Y, Yu H, Zhang H, et al. Hypoxia induces mitochondrial protein lactylation to limit oxidative phosphorylation. Cell Res. 2024;34(1):13–30. 38163844 10.1038/s41422-023-00864-6 PMC10770133

[24] Chen H, Li Y, Li H, Chen X, Fu H, Mao D, Chen W, Lan L, Wang C, Hu K, et al. NBS1 lactylation is required for efficient DNA repair and chemotherapy resistance. Nature. 2024;631(8021):663–669. 38961290 10.1038/s41586-024-07620-9 PMC11254748

[25] Zhu Y, Gu L, Lin X, Liu C, Lu B, Cui K, Zhou F, Zhao Q, Prochownik EV, Fan C, et al. Dynamic regulation of ME1 phosphorylation and acetylation affects lipid metabolism and colorectal tumorigenesis. Mol Cell. 2020;77(1):138–149 e5. 31735643 10.1016/j.molcel.2019.10.015

[26] Gao Q, Zhu H, Dong L, Shi W, Chen R, Song Z, Huang C, Li J, Dong X, Zhou Y, et al. Integrated proteogenomic characterization of HBV-related hepatocellular carcinoma. Cell. 2019;179(2):561–577 e22. 31585088 10.1016/j.cell.2019.08.052

[27] Wan N, Wang N, Yu S, Zhang H, Tang S, Wang D, Lu W, Li H, Delafield DG, Kong Y, et al. Cyclic immonium ion of lactyllysine reveals widespread lactylation in the human proteome. Nat Methods. 2022;19(7):854–864. 35761067 10.1038/s41592-022-01523-1

[28] Wang J, Yang P, Yu T, Gao M, Liu D, Zhang J, Lu C, Chen X, Zhang X, Liu Y. Lactylation of PKM2 suppresses inflammatory metabolic adaptation in pro-inflammatory macrophages. Int J Biol Sci. 2022;18(16):6210–6225. 36439872 10.7150/ijbs.75434 PMC9682528

[29] Nandi S, Razzaghi M, Srivastava D, Dey M. Structural basis for allosteric regulation of pyruvate kinase M2 by phosphorylation and acetylation. J Biol Chem. 2020;295(51):17425–17440. 33453989 10.1074/jbc.RA120.015800 PMC7762928

[30] Gubser PM, Bantug GR, Razik L, Fischer M, Dimeloe S, Hoenger G, Durovic B, Jauch A, Hess C. Rapid effector function of memory CD8+ T cells requires an immediate-early glycolytic switch. Nat Immunol. 2013;14(10):1064–1072. 23955661 10.1038/ni.2687

[31] Yu W, Lei Q, Yang L, Qin G, Liu S, Wang D, Ping Y, Zhang Y. Contradictory roles of lipid metabolism in immune response within the tumor microenvironment. J Hematol Oncol. 2021;14(1):187. 34742349 10.1186/s13045-021-01200-4 PMC8572421

[32] Manzo T, Prentice BM, Anderson KG, Raman A, Schalck A, Codreanu GS, Nava Lauson CB, Tiberti S, Raimondi A, Jones MA, et al. Accumulation of long-chain fatty acids in the tumor microenvironment drives dysfunction in intrapancreatic CD8^+^ T cells. J Exp Med. 2020;217(8): Article e20191920. 32491160 10.1084/jem.20191920 PMC7398173

[33] Peralta RM, Xie B, Lontos K, Nieves-Rosado H, Spahr K, Joshi S, Ford BR, Quann K, Frisch AT, Dean V, et al. Dysfunction of exhausted T cells is enforced by MCT11-mediated lactate metabolism. Nat Immunol. 2024;25(12):2297–2307. 39516648 10.1038/s41590-024-01999-3 PMC11588660

[34] Zhang D, Tang Z, Huang H, Zhou G, Cui C, Weng Y, Liu W, Kim S, Lee S, Perez-Neut M, et al. Metabolic regulation of gene expression by histone lactylation. Nature. 2019;574(7779):575–580. 31645732 10.1038/s41586-019-1678-1 PMC6818755

[35] Susser LI, Nguyen MA, Geoffrion M, Emerton C, Ouimet M, Khacho M, Rayner KJ. Mitochondrial fragmentation promotes inflammation resolution responses in macrophages via histone lactylation. Mol Cell Biol. 2023;43(10):531–546. 37807652 10.1080/10985549.2023.2253131 PMC10569354

[36] Chaudagar K, Hieromnimon HM, Kelley A, Labadie B, Shafran J, Rameshbabu S, Drovetsky C, Bynoe K, Solanki A, Markiewicz E, et al. Suppression of tumor cell lactate-generating signaling pathways eradicates murine PTEN/p53-deficient aggressive-variant prostate cancer via macrophage phagocytosis. Clin Cancer Res. 2023;29(23):4930–4940. 37721526 10.1158/1078-0432.CCR-23-1441 PMC10841690

[37] Li W, Zhou C, Yu L, Hou Z, Liu H, Kong L, Xu Y, He J, Lan J, Ou Q, et al. Tumor-derived lactate promotes resistance to bevacizumab treatment by facilitating autophagy enhancer protein RUBCNL expression through histone H3 lysine 18 lactylation (H3K18la) in colorectal cancer. Autophagy. 2024;20(1):114–130. 37615625 10.1080/15548627.2023.2249762 PMC10761097

[38] Baumgartner CK, Ebrahimi-Nik H, Iracheta-Vellve A, Hamel KM, Olander KE, Davis TGR, McGuire KA, Halvorsen GT, Avila OI, Patel CH, et al. The PTPN2/PTPN1 inhibitor ABBV-CLS-484 unleashes potent anti-tumour immunity. Nature. 2023;622(7984):850–862. 37794185 10.1038/s41586-023-06575-7 PMC10599993

[39] Philips RL, Wang Y, Cheon H, Kanno Y, Gadina M, Sartorelli V, Horvath CM, Darnell JE Jr, Stark GR, O’Shea JJ. The JAK-STAT pathway at 30: Much learned, much more to do. Cell. 2022;185(21):3857–3876. 36240739 10.1016/j.cell.2022.09.023 PMC9815833

[40] Li G, Ma X, Sui S, Chen Y, Li H, Liu L, Zhang X, Zhang L, Hao Y, Yang Z, et al. NAT10/ac4C/JunB facilitates TNBC malignant progression and immunosuppression by driving glycolysis addiction. J Exp Clin Cancer Res. 2024;43(1):278. 39363363 10.1186/s13046-024-03200-x PMC11451012

[41] Liu PS, Chen YT, Li X, Hsueh PC, Tzeng SF, Chen H, Shi PZ, Xie X, Parik S, Planque M, et al. CD40 signal rewires fatty acid and glutamine metabolism for stimulating macrophage anti-tumorigenic functions. Nat Immunol. 2023;24(3):452–462. 36823405 10.1038/s41590-023-01430-3 PMC9977680

[42] Sadik A, Somarribas Patterson LF, Ozturk S, Mohapatra SR, Panitz V, Secker PF, Pfander P, Loth S, Salem H, Prentzell MT, et al. IL4I1 is a metabolic immune checkpoint that activates the AHR and promotes tumor progression. Cell. 2020;182(5):1252–1270 e34. 32818467 10.1016/j.cell.2020.07.038

[43] Wang D, Xu C, Yang W, Chen J, Ou Y, Guan Y, Guan J, Liu Y. E3 ligase RNF167 and deubiquitinase STAMBPL1 modulate mTOR and cancer progression. Mol Cell. 2022;82(4):770–784 e9. 35114100 10.1016/j.molcel.2022.01.002

[44] Wang JM, Gao Q, Zhang Q, Hao L, Jiang JY, Huyan LY, Liu BQ, Yan J, Li C, Wang HQ. Implication of BAG5 downregulation in metabolic reprogramming of cisplatin-resistant ovarian cancer cells via mTORC2 signaling pathway. Biochim Biophys Acta, Mol Cell Res. 2021;1868(9): Article 119076. 34126157 10.1016/j.bbamcr.2021.119076

[45] Yang Y, Han YC, Cao Q, Wang X, Wei XD, Shang MD, Zhang XG, Li X, Hu B, Tian CY, et al. SPOP negatively regulates mTORC1 activity by ubiquitinating Sec13. Cell Signal. 2024;116: Article 111060. 38242269 10.1016/j.cellsig.2024.111060

[46] De S, Holvey-Bates EG, Mahen K, Willard B, Stark GR. The ubiquitin E3 ligase FBXO22 degrades PD-L1 and sensitizes cancer cells to DNA damage. Proc Natl Acad Sci USA. 2021;118(47): Article e2112674118. 34795058 10.1073/pnas.2112674118 PMC8617495

[47] Ruan H, Bao L, Tao Z, Chen K. Flightless I homolog reverses enzalutamide resistance through PD-L1-mediated immune evasion in prostate cancer. Cancer Immunol Res. 2021;9(7):838–852. 34011528 10.1158/2326-6066.CIR-20-0729

[48] Du Y, Lyu Y, Lin J, Ma C, Zhang Q, Zhang Y, Qiu L, Tan W. Membrane-anchored DNA nanojunctions enable closer antigen-presenting cell-T-cell contact in elevated T-cell receptor triggering. Nat Nanotechnol. 2023;18(7):818–827. 36894782 10.1038/s41565-023-01333-2

[49] Chen Y, He J, Chen R, Wang Z, Dai Z, Liang X, Wu W, Luo P, Zhang J, Peng Y, et al. Pan-cancer analysis of the immunological role of PDIA5: A potential target for immunotherapy. Front Immunol. 2022;13: Article 881722. 36003400 10.3389/fimmu.2022.881722 PMC9393377

[50] Zhang H, He J, Dai Z, Wang Z, Liang X, He F, Xia Z, Feng S, Cao H, Zhang L, et al. PDIA5 is correlated with immune infiltration and predicts poor prognosis in gliomas. Front Immunol. 2021;12: Article 628966. 33664747 10.3389/fimmu.2021.628966 PMC7921737

[51] Song J, Wei R, Huo S, Liu C, Liu X. Versican enrichment predicts poor prognosis and response to adjuvant therapy and immunotherapy in gastric cancer. Front Immunol. 2022;13: Article 960570. 36203562 10.3389/fimmu.2022.960570 PMC9530562

[52] Guo J, Sun D, Zhang J, Guo J, Wu Z, Chen Y, Xu Y, Zhou D, Cui Y, Mo Q, et al. The E3 ubiquitin ligase RBCK1: Implications in the tumor immune microenvironment and antiangiogenic therapy of glioma. Comput Struct Biotechnol J. 2023;21:5212–5227. 37928949 10.1016/j.csbj.2023.10.020 PMC10624590

[53] Park SA, Sung NJ, Choi BJ, Kim W, Kim SH, Surh YJ. Gremlin-1 augments the oestrogen-related receptor α signalling through EGFR activation: Implications for the progression of breast cancer. Br J Cancer. 2020;123(6):988–999. 32572171 10.1038/s41416-020-0945-0 PMC7493948

[54] Sung NJ, Kim NH, Surh YJ, Park SA. Gremlin-1 promotes metastasis of breast cancer cells by activating STAT3-MMP13 signaling pathway. Int J Mol Sci. 2020;21(23):9227. 33287358 10.3390/ijms21239227 PMC7730512

[55] Baboota RK, Rawshani A, Bonnet L, Li X, Yang H, Mardinoglu A, Tchkonia T, Kirkland JL, Hoffmann A, Dietrich A, et al. BMP4 and Gremlin 1 regulate hepatic cell senescence during clinical progression of NAFLD/NASH. Nat Metab. 2022;4(8):1007–1021. 35995996 10.1038/s42255-022-00620-x PMC9398907

[56] Shah R, Singh SJ, Eddy K, Filipp FV, Chen S. Concurrent targeting of glutaminolysis and metabotropic glutamate receptor 1 (GRM1) reduces glutamate bioavailability in GRM1^+^ melanoma. Cancer Res. 2019;79(8):1799–1809. 30987979 10.1158/0008-5472.CAN-18-1500 PMC6469683

[57] Krug K, Mertins P, Zhang B, Hornbeck P, Raju R, Ahmad R, Szucs M, Mundt F, Forestier D, Jane-Valbuena J, et al. A curated resource for phosphosite-specific signature analysis. Mol Cell Proteomics. 2019;18(3):576–593. 30563849 10.1074/mcp.TIR118.000943 PMC6398202

[58] Zheng B, Pan Y, Qian F, Liu D, Ye D, Yu B, Zhong S, Zheng W, Wang X, Zhou B, et al. High sugar induced RCC2 lactylation drives breast cancer tumorigenicity through upregulating MAD2L1. Adv Sci. 2025;12(21): Article e2415530. 10.1002/advs.202415530 PMC12140329 40145796

[59] De Leo A, Ugolini A, Yu X, Scirocchi F, Scocozza D, Peixoto B, Pace A, D’Angelo L, Liu JKC, Etame AB, et al. Glucose-driven histone lactylation promotes the immunosuppressive activity of monocyte-derived macrophages in glioblastoma. Immunity. 2024;57(5):1105–1123 e8. 38703775 10.1016/j.immuni.2024.04.006 PMC11114377

[60] Kao KC, Vilbois S, Tsai CH, Ho PC. Metabolic communication in the tumour-immune microenvironment. Nat Cell Biol. 2022;24(11):1574–1583. 36229606 10.1038/s41556-022-01002-x

[61] Zak J, Pratumchai I, Marro BS, Marquardt KL, Zavareh RB, Lairson LL, Oldstone MBA, Varner JA, Hegerova L, Cao Q, et al. JAK inhibition enhances checkpoint blockade immunotherapy in patients with Hodgkin lymphoma. Science. 2024;384(6702):eade8520. 38900864 10.1126/science.ade8520 PMC11283877

[62] Mathew D, Marmarelis ME, Foley C, Bauml JM, Ye D, Ghinnagow R, Ngiow SF, Klapholz M, Jun S, Zhang Z, et al. Combined JAK inhibition and PD-1 immunotherapy for non-small cell lung cancer patients. Science. 2024;384(6702):eadf1329. 38900877 10.1126/science.adf1329 PMC11327955

[63] Wang C, Xu H, Lin S, Deng W, Zhou J, Zhang Y, Shi Y, Peng D, Xue Y. GPS 5.0: An update on the prediction of kinase-specific phosphorylation sites in proteins. Genomics Proteomics Bioinformatics. 2020;18(1):72–80. 32200042 10.1016/j.gpb.2020.01.001 PMC7393560

[64] Wiredja DD, Koyuturk M, Chance MR. The KSEA app: A web-based tool for kinase activity inference from quantitative phosphoproteomics. Bioinformatics. 2017;33(21):3489–3491. 28655153 10.1093/bioinformatics/btx415 PMC5860163

