# Supplementary material for: Integrated Proteogenomic Characterization Identifies Breast Cancer Immune Subgroups and Subtype-Specific Therapeutic Strategies
Source: Research (Wash D C). 2026 May 12;9:1271. doi: 10.34133/research.1271 (PMC13161541; doi:10.34133/research.1271)
Supplement: Supplementary 1 — Figs. S1 to S5 Tables S1 to S11 [file research.1271.f1.zip › Supplementary Legends.docx]

**Supplementary Figures**

Figure S1 The validation of immune subtypes.

(A-D) Based on the PDC000120 and PDC000173 from CPTAC (Clinical Proteomic Tumor Analysis Consortium) database, performing the proteomic immunophenotyping method and GSEA enrichment analysis. (E) Multiple immune cells abundance estimated from Xcell, and the bar chart displays the immune cell infiltration of three immune clusters. (F) Spearman correlation between the GSVA values for each sample of the MSigDB hallmark genesets and the EXHAUSTED CD8_TCELL and CD8_TCELL abundance. (G) Sankey diagram illustrating the distribution and transition patterns between immune activity states (IA, IR, and IS) and tumor microenvironment subtypes, including Depleted, Fibrotic, Immune_Enriched_Fibrotic, and Immune_Enriched_non_Fibrotic. The width of the flows represents the relative proportion of samples assigned to each category. (H) Forest plot of univariate Cox proportional hazards analysis evaluating the associations between clinicopathological variables, immune typing, and survival. Hazard ratios (HRs) with 95% confidence intervals (CIs) are shown for each variable. (I-K) Proteomic KEGG enrichment analysis displays key oncogenic pathways upregulated and downregulated in three immune subtypes. (L) Bar chart displaying highly expressed proteins in three immune subtypes.

Figure S2. Genomic landscape of the breast cancer subtypes.

(A)Genic mutation frequency of *PIK3CA/TP53*/*KMT2C*. (B) The TMB in three subtypes. (C-E) Integrative analysis of the two databases revealed how TP53, PIK3CA and *KMT2C* mutations shape survival in the IR, IA and IS subtypes, respectively. (F-H) GSEA enrichment analysis between *PIK3CA/TP53*/*KMT2C*-WT and *PIK3CA/TP53*/*KMT2C*-Mut.

Figure S3. The kinase prediction of ALDOA_S46, ALDOA_S36, and PKM_S37

(A) The GPS (Group-based Prediction System) 5.0 predicts the kinases of ALDOA_S46, ALDOA_S36, and PKM_S37. (B) KEGG enrichment analysis on the differentially expressed genes between the high- and low-abundance ALDOA_S46 groups. (C) GEPIA predicts MAPK and PI3K-AKT related proteins that interact with ALDOA. (D) The bar chart displays the GSEA enrichment analysis results of immune IS subtypes in the lactylation and phosphorylation modified groups compared to other subtypes. Red indicates upregulation, blue indicates downregulation, and the bar length is significant. Q value is indicated by asterisks. (E) Volcano plot showing differential lactylation between the IS subtype and the other immune clusters. Lactylation sites on triglyceride catabolism and glycolysis proteins are highlighted. (F-G) Bubble plot representing significant correlations between triglyceride catabolism and glycolysis lactylation sites and protein levels from the IFN-γ pathway. P values were derived from Spearman’s correlation test.

Figure S4. The effects of NAA15 on proliferation, metabolism and survival of breast cancer

(A-B) Significant associations between core histone lactylation sites and acyltransferase, deacylases, and bromodomain-containing proteins in IR and IS tumors. (C) Kaplan-Meier analysis of overall survival according to NAA15 protein expression. (D) The genes related to proliferation and metabolism protein expression between NAA15-high and NAA15-low. (E-F) Kaplan-Meier analysis of overall survival according to NAA15 and NAA 25 protein expression based on the data from TCGA, Cell 2015. (G) Volcano plot was used to display the differentially expressed genes between the high- and low-H1 lactylation groups. (H) Density distribution curve was used to show the distribution of histone H1 lactylation signature gene scores. (I) The correlation between immune infiltration levels and H1 lactylation signature scores.

Figure S5. The expression of three subtypes of markers and the functions of differential phosphorylation and lactylation sites.

(A) Receiver operating characteristic curves for using the protein expression of breast cancer-associated genes to identify the corresponding subtypes. (B) Receiver operating characteristic curves for using the protein expression of breast cancer-associated genes to identify the corresponding subtypes by using PDC000173 dataset. (C) Based on RNA-seq, the expression levels of three immune detection points in three immune clusters.

**Supplementary tables**

Table S1 Sample information

Table S2 Heatmap scaledata

Table S3 PDC000173-108sample

Table S4 PDC000120-125sample

Table S5 T cells abundance and hallmaker gene sets

Table S6 Sample mutation status

Table S7 Prediction of PKM and ALDOA phosphate kinases

Table S8 Histone lactylation in different subtypes

Table S9 Glycometabolism genes and histone lactylation

Table S10 The abundance of immune cells based on the Xcell

Table S11 The prediction of immunotherapy efficacy
